# Implementation of an electronic care pathway for hip fracture patients: a pilot before and after study

**DOI:** 10.1186/s12891-020-03834-w

**Published:** 2020-12-11

**Authors:** Jason Talevski, Viviana Guerrero-Cedeño, Oddom Demontiero, Pushpa Suriyaarachchi, Derek Boersma, Sara Vogrin, Sharon Brennan-Olsen, Gustavo Duque

**Affiliations:** 1 Australian Institute for Musculoskeletal Science (AIMSS), The University of Melbourne and Western Health, Melbourne, VIC Australia; 2 Department of Medicine-Western Health, The University of Melbourne, Melbourne, VIC Australia; 3 Department of Geriatric Medicine, Nepean Hospital, Penrith, NSW Australia

**Keywords:** Care pathways, Older adults, Orthogeriatric, Hip fracture

## Abstract

**Background:**

Care pathways are generally paper-based and can cause communication failures between multidisciplinary teams, potentially compromising the safety of the patient. Computerized care pathways may facilitate better communication between clinical teams. This study aimed to investigate whether an electronic care pathway (e-pathway) reduces delays in surgery and hospital length of stay compared to a traditional paper-based care pathway (control) in hip fracture patients.

**Methods:**

A single-centre evaluation with a retrospective control group was conducted in the Orthogeriatric Ward, Nepean Hospital, New South Wales, Australia. We enrolled patients aged > 65 years that were hospitalized for a hip fracture in 2008 (control group) and 2012 (e-pathway group). The e-pathway provided the essential steps in the care of patients with hip fracture, including examinations and treatment to be carried out. Main outcome measures were delay in surgery and hospital length of stay; secondary outcomes were in-hospital mortality and discharge location.

**Results:**

A total of 181 patients were enrolled in the study (129 control; 54 e-pathway group). There was a significant reduction in delay to surgery in the e-pathway group compared to control group in unadjusted (OR = 0.19; CI 0.09–0.39; *p* < 0.001) and adjusted (OR = 0.22; CI 0.10–0.49; *p* < 0.001) models. There were no significant differences between groups for length of stay (median 11 vs 12 days; *p* = 0.567), in-hospital mortality (1 vs 7 participants; *p* = 0.206) or discharge location (*p* = 0.206).

**Conclusions:**

This pilot study suggests that, compared to a paper-based care pathway, implementation of an e-pathway for hip fracture patients results in a reduction in total number of delays to surgery, but not hospital length of stay. Further evaluation is warranted using a larger cohort investigating both clinical and patient-reported outcome measures.

## Background

Hip fracture rates have continued to decrease over the last two decades in countries including the USA [1], Canada [2], Australia [3] and Scandinavia (Denmark, Norway, and Sweden) [4], although the absolute number of hip fractures has increased most likely due to aging populations and greater number of persons at risk [4, 5]. Hip fractures are the most severe type of fracture posing an important risk for in-hospital complications [6] and burden on quality of life [7, 8]. The mortality rate in the first year following a hip fracture is 25% in adults aged > 70 years, with an increased risk of mortality persisting for at least 5 years following the fracture [9]. Additionally, approximately 50% of people become physically disabled for the remainder of their life and require assistance with basic activities of daily living such as dressing or toileting [10, 11].

Hospitals often have care protocols for post-hip fracture management that are commonly referred to as “Orthogeriatric Services”. These care pathways provide orthopaedic and geriatric co-care of patients admitted to hospital with hip fractures, focusing on expediting surgery, ensuring optimal management and adherence to a care plan, and delivery of secondary fracture prevention through osteoporosis management and falls prevention [12]. Care pathways have demonstrated to be effective in preventing common post-discharge complications (e.g. deep venous thrombosis, surgical site infection, pressure ulcers) [13]; recovery of basic activities of daily living [14, 15] and quality of life [16, 17]; and decreased mortality [18] and re-fracture rates [19]. However, care pathways are generally paper based, which can often cause communication failures between clinical teams, potentially causing delays in surgery and compromising patient safety [20, 21].

Electronic ‘computerized’ care pathways (referred to as “e-pathways” from here forth) promote and facilitate better communication between multidisciplinary teams and can potentially lead to better health outcomes for patients. An e-pathway can be defined as a computerized care pathway that guides health care professionals with evidence-based treatment plans, while allowing messages between a multidisciplinary team to be exchanged within the electronic system [22]. Studies evaluating the effectiveness of e-pathways among hip fracture patients is limited. One pre-post study in older adults with hip fracture found no significant differences in delirium rates, mean length of stay, falls or discharges to long-term care after using an e-pathway compared to standard care [23]. Another study found that an electronic referral system resulted in greater delivery of osteoporosis services and significant improvements in the management of osteoporosis in patients with hip fracture, compared to a non-intervention control group [24].

The primary aim of this pilot study is to evaluate the effect of an e-pathway on patient delays to surgery (> 48 h post-admission) and hospital length of stay in hip fracture patients, compared to a traditional paper-based care pathway (control group).

## Methods

### Study design

A single-centre evaluation with a retrospective control group. Outcomes were compared in patients from two time periods: 2008 (before e-pathway implementation) and 2012 (after e-pathway implementation). Ethical approval to conduct the study was obtained from the Nepean Blue Mountains Human Research Ethics Committee (SSA/15/Nepean/17).

### Participants and setting

A formal sample size calculation was not required or undertaken as this was a pilot study designed to primarily provide data for a larger definitive efficacy trial [25]. Patients aged > 65 years that were hospitalized in the Orthogeriatric Ward of the Nepean Hospital, New South Wales, Australia in 2008 and 2012 with a hip fracture were included for participation. Patients were identified by searching the hospital medical records database using International Classification of Diseases (ICD-10) codes. Participant characteristics including age, sex, living status prior to fracture, fracture type and surgery type were collected.

### Fractured neck of femur checklist (control group)

The ‘Orthogeriatric Model of Care: Clinical Practice Guide’ was developed by the New South Wales Agency for Clinical Innovation in 2010 [26]. This model of care aims to provide a clear and practical guide for caring for orthopaedic patients and ensure all patients with a hip fracture (aged > 65 years) consistently receive best practice clinical care by an orthopaedic surgeon and geriatrician from the time of admission. The model of care is driven by a ‘Fractured Neck of Femur Checklist’ that provides the essential steps in the care of patients with hip fracture, guiding the multidisciplinary team on examinations, tests and treatment to be carried out according to each patient phase (preoperative and postoperative).

### Orthogeriatric preoperative and postoperative checklist (E-pathway)

An electronic format of the ‘Fractured Neck of Femur Checklist’ was developed using Cerner electronic medical record Power Chart (Cerner, North Kansas City, MO, USA), replicating the original questions included in the paper based New South Wales Agency for Clinical Innovation guidelines [26] – this was named the ‘Orthogeriatric Preoperative and Postoperative Checklist’. This clinical portal allowed clinicians to access a range of patient medical information including general observations, test results, medications and other relevant patient information from a single electronic window. All clinicians and nurses involved in the study were notified of the implementation of the e-pathway and received education and training in its use. As participating health professionals were familiar with Power Chart, a 1-h training session was provided to staff explaining how to navigate between the several screens associated with this e-pathway. The format and treatment questions comprising the e-pathway are detailed in Appendix 1.

### Outcomes

The primary outcomes were delay to surgery (> 48 h) and hospital length of stay. Secondary outcomes included in-hospital mortality and discharge location.

### Statistical analysis

Descriptive statistics were calculated for demographic variables and summarized as mean (SD) or frequency (percentage). The differences between the two groups were compared using Mann–Whitney U-tests and independent t-tests for continuous data, or chi-squared tests for categorical data. Outcomes were analyzed using logistic and liner regression models for categorical and continuous variables, respectively. Variables which were significantly different between groups were added to multivariable regression models. A *p*-value of < 0.05 was considered statistically significant. All statistical analyses were performed using STATA (version 16) statistical software.

## Results

### Participants

A total of 181 patients were recruited into the study, 129 during the 2008 period and 52 during the 2012 period. The mean age of patients was 84.3 years, majority of patients were female (72.9%) and over half the patients were living at home before admission (61.1%). Kidney function (eGFR) and age were significantly higher in the control group, and therefore were adjusted for in the analysis. Patient characteristics are shown in Table 1.

**Table 1:** Demographic characteristics of patients

	Control Group (***n*** = 129)	E-pathway Group (***n*** = 52)	***P***-value
**Age, mean (SD)**	84.9 (5.9)	82.8 (7.2)	**0.036**
**Sex, n (%)**			
Male	35 (27.1%)	14 (27.0%)	
Female	94 (72.9%)	38 (73.0%)	0.977
**Living Status Before Admission, n (%)**			
Home	74 (57.4%)	33 (63.5%)	
Care Facility/Residential Aged Care	55 (42.6%)	19 (36.5%)	0.086
**Fracture Type, n (%)**			
Intracapsular	63 (48.8%)	20 (38.0%)	
Intertrochanteric	55 (42.6%)	13 (25.0%)	
Subtrochanteric	10 (8.8%)	2 (4.0%)	
Unknown	1 (0.8%)	17 (33.0%)	0.695
**Type of Surgery, n (%)**			
Internal Fixation (cannulated screws)	3 (2.3%)	4 (8.0%)	
Hemiarthroplasty	45 (34.9%)	21 (40.0%)	
Total Hip Replacement	53 (41.1%)	14 (27.0%)	
Sliding Hip Screws/DHS	27 (20.9%)	8 (15.0%)	
Intramedullary Nails/Gamma	1 (0.8%)	0 (0.0%)	
Other	0 (0.0%)	5 (10.0%)	0.201
**eGFR, mean (SD)**	50.7 (11.7)	66.4 (18.8)	**< 0.001**

### Primary outcomes

77 patients (59.7%) in the control group had a delayed surgery of > 48 h after admission compared to 11 patients (21.6%) in the e-pathway group. This difference was statistically significant in unadjusted (OR = 0.19; CI 0.09–0.39; *p* < 0.001) and adjusted (OR = 0.22; CI 0.10–0.49; *p* < 0.001) models. There were no significant differences between groups for length of stay (median 12 vs 11 days; *p* = 0.567). Unadjusted and adjusted results are shown in Table 2.

**Table 2:** Primary and secondary outcomes before and after implementation of the e-pathway

	Control Group (***n*** = 129)	E-pathway Group (***n*** = 52)	Unadjusted		Adjusted^**a**^	
			OR (95% CI)	***P***-value	OR (95% CI)	***P***-value
**Primary Outcomes**						
Delay in surgery (> 48 h), n (%)	77 (59.7%)	11 (21.6%)	**0.19 (0.09–0.39)**	**< 0.001**	**0.22 (0.10–0.49)**	**< 0.001**
LOS, median (IQR)	12 (7–19)	11 (7–17)	−1.12 (−4.41–2.18)^b^	0.505	−1.07 (−4.77–2.62)^b^	0.567
**Secondary Outcomes**						
In-hospital Mortality, n (%)	7 (5.4%)	1 (1.9%)	0.34 (0.04–2.85)	0.321	0.19 (0.01–2.49)	0.206
Discharge Location, n (%)						
Home	11 (8.5%)	6 (11.5%)	1.40 (0.49–4.00)	0.531	1.52 (0.40–5.73)	0.539
Nursing Home/RAC	32 (24.8%)	9 (17.3%)	0.63 (0.28–1.44)	0.278	0.67 (0.27–1.67)	0.393

^a^Adjusted for age and estimated glomerular filtration rate (eGFR)

^b^Beta-coefficient (Confidence Intervals)

*OR* Odds Ratio, *CI* Confidence Interval, *SD* Standard Deviation, *IQR* Interquartile Range

### Secondary outcomes

Implementation of the e-pathway had no impact on in-hospital mortality and discharge location compared to control group participants. Unadjusted and adjusted results are shown in Table 2.

SD = Standard Deviation; eGFR = Estimated Glomerular Filtration Rate.

## Discussion

The benefits of electronic care pathways for the management of hip fracture patients is not clear, as previous studies have primarily focused on evaluating paper-based care pathways. This pilot study aimed to not only reduce this knowledge gap, but also provide useful information to inform future research within this area. Our hypothesis was confirmed that an e-pathway has the potential to reduce delays in surgery compared to a traditional paper-based care pathway. Patients in the e-pathway group had a significantly lower amount of surgery delays compared to the control group. There were no differences between groups in hospital length of stay, in-hospital mortality and discharge location.

There is a plethora of literature that highlights the effectiveness of paper-based clinical care pathways in hip fracture patients across multiple health outcomes [14, 17–19, 27], while studies evaluating e-pathways among hip fracture patients is limited [23, 24]. Although paper-based care pathways have evolved and adapted with the changes with our complex and ever-changing healthcare system, they have received some criticism in terms of limitations of efficient communication between multidisciplinary teams [28]. Throughout the patient journey, multiple patient documents are created at each stage including the initial admission encounter, inpatient care (pre and post-operative), transfers of care, referrals to specialist services and discharge care; and can often lead to inefficient information mangement. Instances of missed information could contribute to potential adverse events and patient harm. The development and implementation of e-pathways can be set within today’s context of technology and may overcome these limitations. Furthermore, the computerization of paper-based care pathways is inevitable as many hospitals have adopted to the use of electronic medical records [29], which provides a new opportunity for care pathways to be integrated within this system. Given that the literature for e-pathways in hip fracture patients is limited, further research is warranted in the implementation, effectiveness, and sustainability of computerising paper-based care pathways.

Timing of surgery post-hip fracture is thought to play an important role regarding survival, with clinical practice guidelines recommending surgical treatment of hip fracture within 48 h of admission [30, 31]. A recent review of 28 observational studies reported that patients who were operated on within 48 h were associated with 20% lower risk of 12-month mortality and fewer post-operative complications (8% vs. 17%) [32]. Timing of surgery for hip fracture patients remains a challenge in hospitals because of a mix of patient and organizational barriers [33]. System-levels factors associated with delays to surgery after hip fracture include surgical readiness, available resources, lack of communication between multidisciplinary staff and patient out-of-hours admission [34]. Previous studies have shown that electronic care systems promote greater multidisciplinary involvement and improve interdisciplinary communication [35, 36]. In this study, the lower number of patients with delays in surgery in the e-pathway group compared to the paper-based control group may be an indicator of improved communication and coordination between clinicians and surgeons. Therefore, use of electronic care pathways have the potential to improve communication between multidisciplinary teams to ensure timely and appropriate care post-hip fracture.

Systematic reviews evaluating the effectiveness of clinical care pathways have shown mixed results regarding decreased hospital length of stay and in-hospital mortality compared to usual hip fracture care [14, 18, 27]. It was therefore not surprising that these outcomes did not differ between groups in this study. The link between timing of surgery and length of stay has not been specifically examined, though the link between timing of surgery and fewer post-operative complications has been [32], which should possibility translate to reduced hospital length of stay. However, length of stay is a difficult concept to interpret as a quality criterion for hip fracture patient care. Although decreasing length of hospital stay is proof of cost-effectiveness for care pathways, it does not provide any information on quality of treatment or patient outcomes [37]. The effect of e-pathways on hospital length of stay and in-hospital mortality in hip fracture patients should be investigated in a larger study.

Finally, although clinicians and nurses involved in the study were trained in the use of the e-pathway, which is an essential implementation strategy when introducing a new intervention, training alone is not sufficient to effect ongoing change and uptake into clinical practice [38]. Successful implementation normally requires an implementation plan and a multifaceted approach that includes collaboration between stakeholders and health services, staff flexibility, and a culture receptive to change [39]. A recent implementation study identified five strategies which may contribute to the successful implementation of an electronic care pathway: a strong national policy context for the rationalization of processes and data collection of efficiency indicator targets; financial and organisational resources; multidisciplinary engagement; guidelines and documentation for the standardisation and implementation; and development of an implementation protocol based on national guidelines and clinical expertise [40]. Future research in electronic care pathways should be guided by these strategies in order to achieve successful implementation and integration into routine practice.

### Limitations

This study has several limitations that should be acknowledged. Although the addition of a control group strengthens our findings, the retrospective design can lead to an underestimation of the effect between groups due to other influences such as changes to ward structures, staffing changes and processes between the periods examined. Therefore, the results in our outcomes of interest may have occurred without implementation of the e-pathway. Despite recruitment of the two patient samples within the same hospital, we did find the control group was older and had lower kidney function, suggesting more frailty in this group. However, we adjusted for these differences to determine the independent effects of the e-pathway on primary and secondary outcomes. We were unable to comment on patient factors known to be associated with delays in surgery after hip fracture such as frailty, comorbidity and socioeconomic status [33], as this data were not collected as part of the study. Finally, we did not assess implementation fidelity of the e-pathway, so it is unclear as to whether the e-pathway was implemented in practice as it was intended to.

## Conclusions

It is claimed that electronic care pathways enable a superior way of working that is not possible in a paper-based environment, however, data to back this up is limited in hip fracture care. This pilot study suggests that implementation of an electronic clinical care pathway for hip fracture patients has the potential to reduce delays in surgery compared to a traditional paper-based care pathway. A future trial to confirm these effects should be randomized, recruit a larger cohort of participants to be adequately powered to detect significant change; include both biomedical-oriented (e.g. length of stay, mortality) and objective (physical function, quality of life) outcome measures; and include a cost-effectiveness evaluation.

## Supplementary Information

**Additional file 1.**

## Data Availability

Data sharing is not applicable to this article as no datasets were generated or analysed during the current study.

## Abbreviations

ICD-10: International Classification of Diseases
eGFR: Estimated Glomerular Filtration Rate
USA: United States of America
OR: Odds Ratio
SD: Standard Deviation
CI: Confidence Interval
