# Supplementary material for: Implementation of an electronic care pathway for hip fracture patients: a pilot before and after study
Source: BMC Musculoskelet Disord. 2020 Dec 11;21:837. doi: 10.1186/s12891-020-03834-w (PMC7731480; doi:10.1186/s12891-020-03834-w)

***
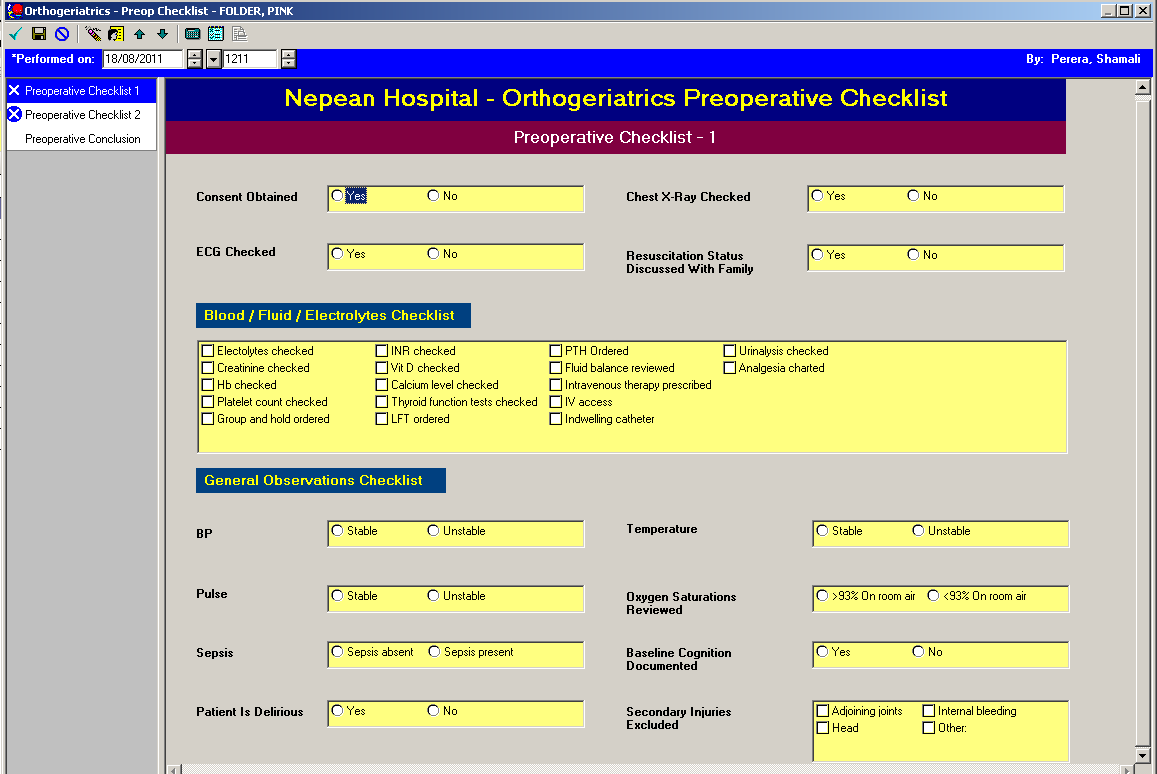
*Pre-operative Checklist (Section 1)**

**
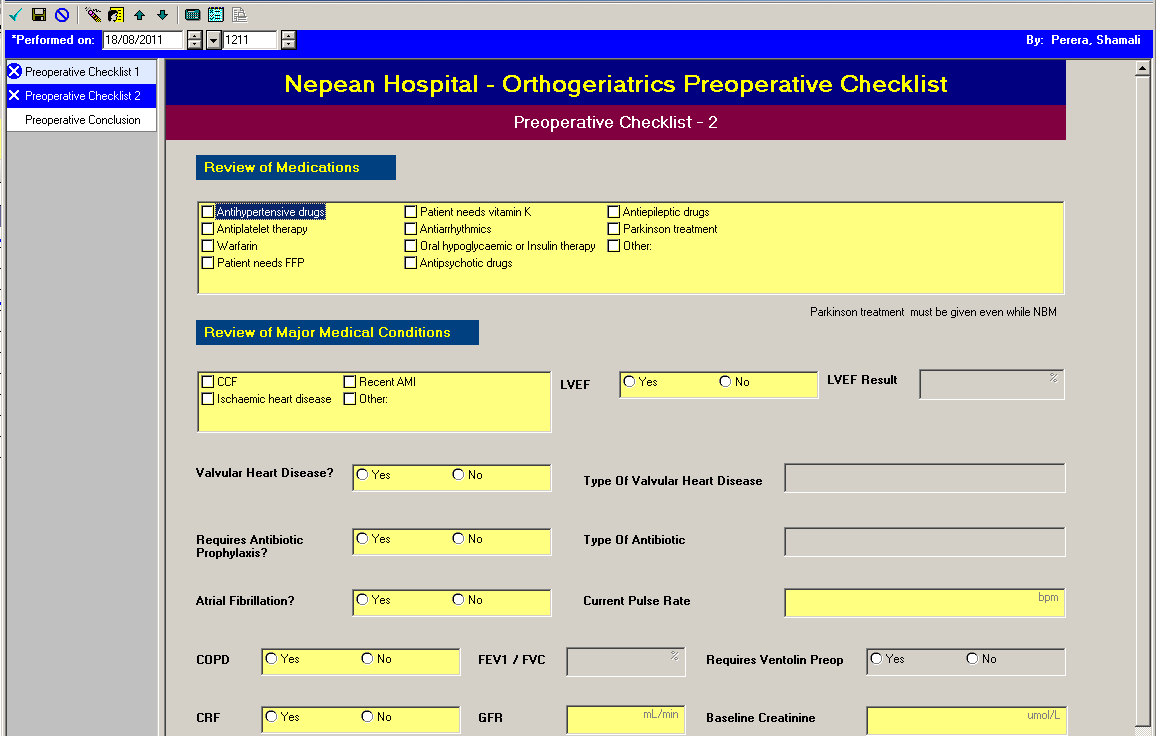
 Pre-operative Checklist (Section 2)**

- **Yellow indicates required/mandatory fields**
- **The Powerform cannot be signed off until data is entered**

**Greyed out fields are activated when “Yes” is selected in the previous box**

**Pre-operative Checklist (Section 3)**


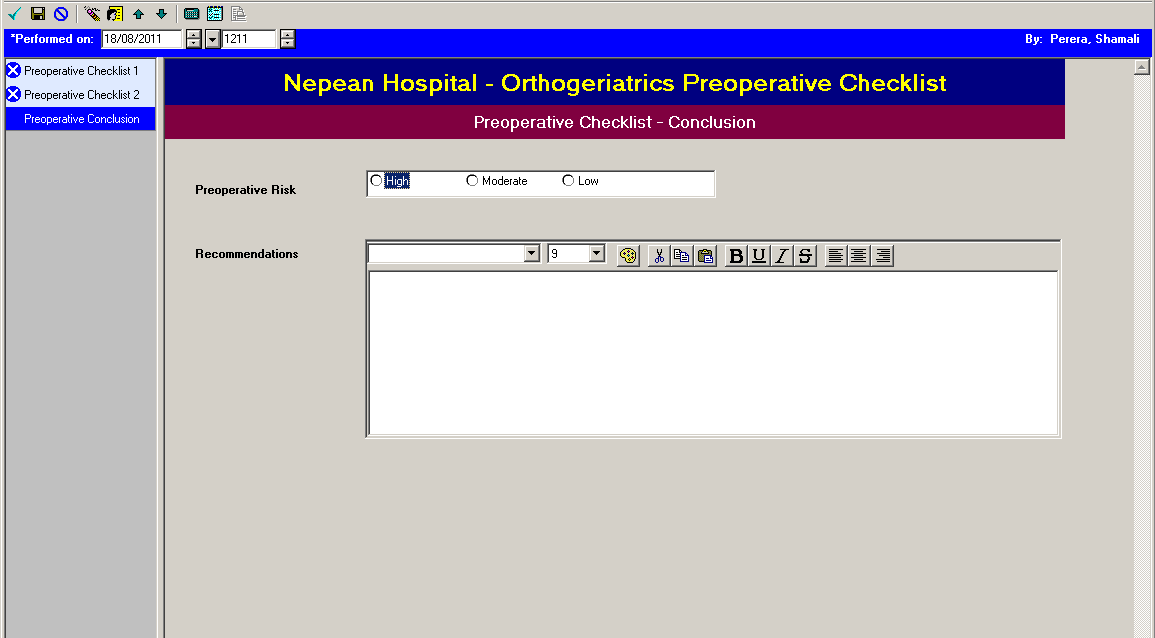


**Post-operative Checklist (Section 1)**


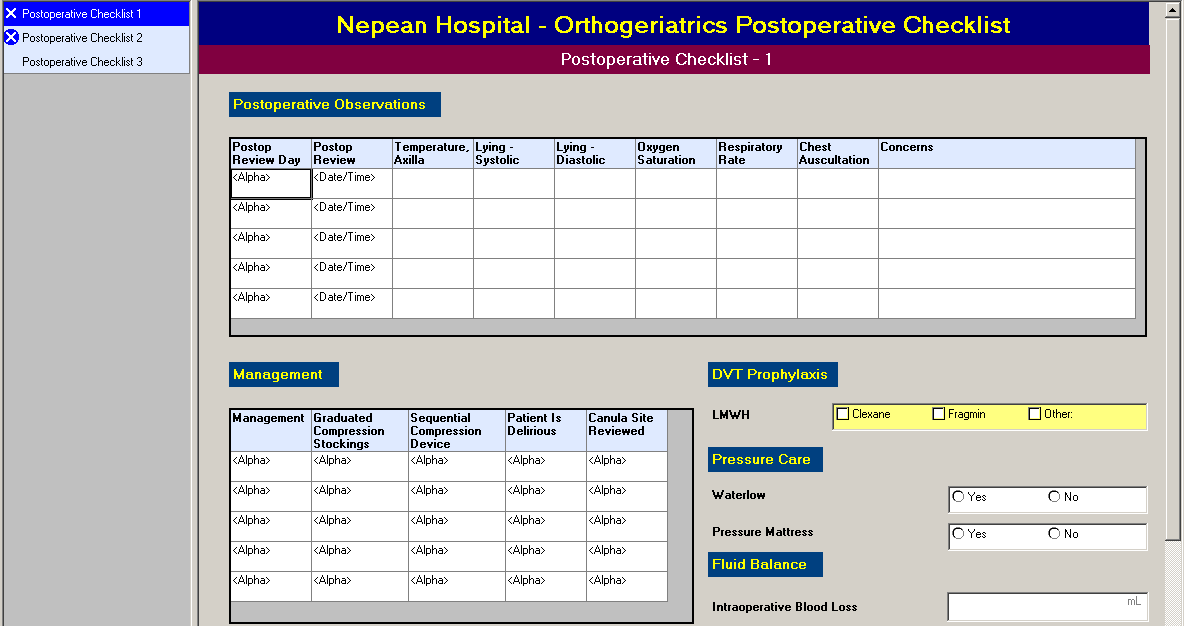

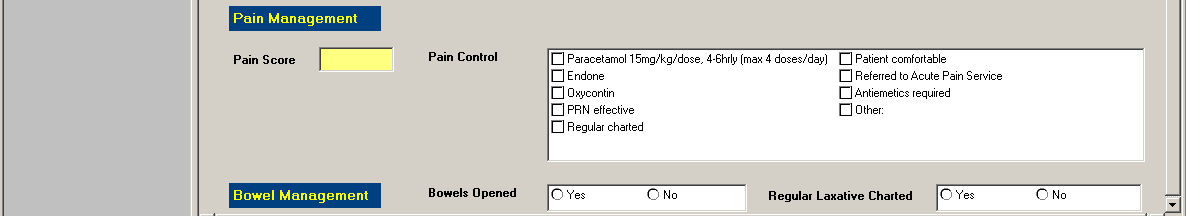


**Post-operative Checklist (Section 2)**


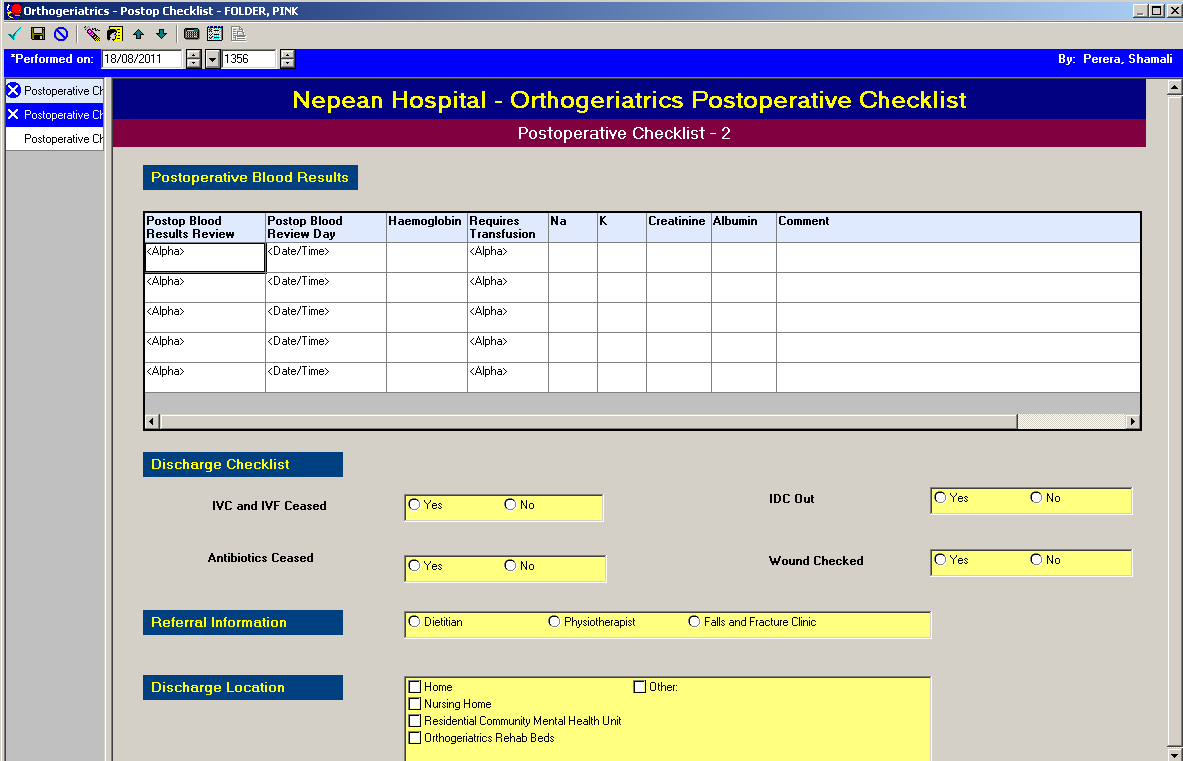


**Post-operative Checklist (Section 3)**


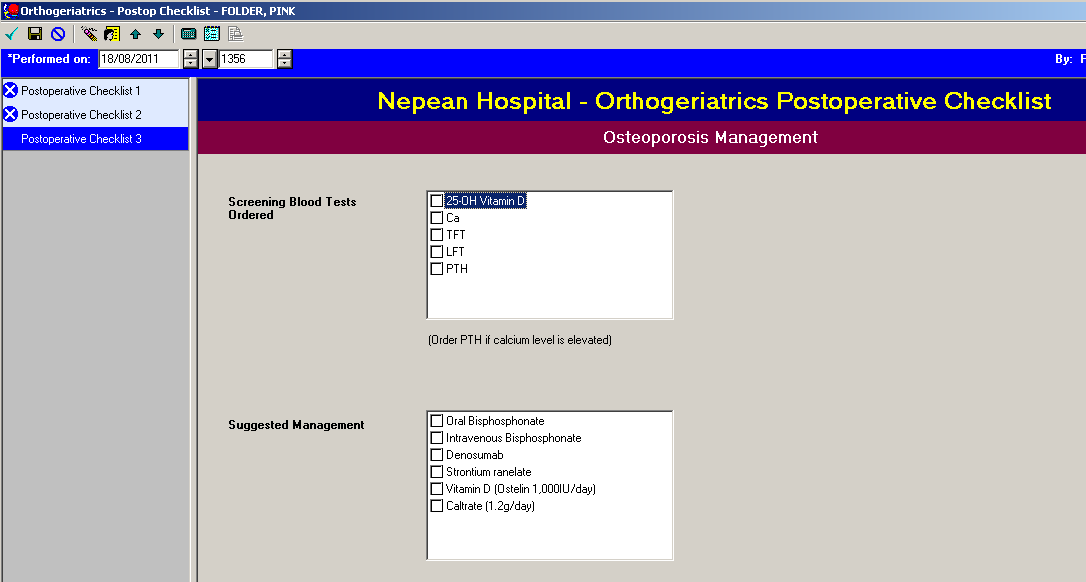

Supplement: Supplementary file 1 — Additional file 1. [file 12891_2020_3834_MOESM1_ESM.docx]
